# Mapping Molecular Determinants of Antigenicity and Pathogenicity of Infectious Bursal Disease Virus (IBDV): A Scoping Review

**DOI:** 10.3390/v18050489

**Published:** 2026-04-23

**Authors:** Francesca Romana Tonellato, Francesca Poletto, Cristina Andolfatto, Claudia Maria Tucciarone, Giovanni Franzo, Mattia Cecchinato, Matteo Legnardi

**Affiliations:** 1 Department of Animal Medicine, Production and Health (MAPS), University of Padua, 35020 Legnaro, Italy; 2 Department of Comparative Biomedicine and Food Science (BCA), University of Padua, 35020 Legnaro, Italy

**Keywords:** infectious bursal disease virus, molecular determinant, antigenicity, pathogenicity, genetic marker, amino acid substitution, viral evolution, scoping review

## Abstract

Infectious bursal disease virus (IBDV) is an immunosuppressive pathogen posing a major threat to poultry health worldwide. Its marked phenotypic variability is driven by the rapid evolution of its double-stranded RNA genome, primarily achieved through mutation and reassortment. Although extensive evidence has been generated on molecular determinants of antigenicity and pathogenicity, interpretation is often hindered by heterogeneity and lack of systematicity. This scoping review synthesizes over 35 years of research on amino acid positions influencing IBDV phenotype. A total of 62 studies reporting 107 functionally relevant sites were identified and critically appraised based on evidence type, methodological approach, and ability to infer causality. The results confirmed the central role of VP2, particularly its hypervariable region, while also highlighting the increasingly recognized contribution of other viral proteins. Despite good agreement, comparability across studies was limited by substantial heterogeneity in experimental design and the frequent focus on partial genomic regions. Notably, some molecular markers were context-dependent or inconsistently associated with phenotypic outcomes, underscoring the need for proper interpretation of molecular determinants and for more standardized and comprehensive approaches, including full-genome analyses and reverse genetics. Overall, these findings provide a valuable framework for enhancing molecular diagnostics and supporting the rational design of next-generation vaccines.

## 1. Introduction

Infectious bursal disease (IBD) is one of the most impactful viral diseases of chickens, characterized by high morbidity and a variable degree of mortality. Its clinical presentation is typically characterized by non-specific signs such as prostration, anorexia and diarrhea, along with lesions in immune organs and hemorrhages in the thigh and pectoral muscles. The most remarkable consequence of IBD is the depletion of developing B lymphocytes within the bursa of Fabricius, thus resulting in immunosuppression [1]. Acute IBD forms are mostly seen in chickens aged between 3 and 6 weeks, when the bursa is at the peak of its functionality [2], whereas earlier infections tend to be subclinical and older birds become resistant as the bursa undergoes physiological involution [3]. Nonetheless, IBD manifestation also depends on factors such as the chicken breed, immune status, presence of intercurrent conditions and, perhaps most importantly, the viral strain involved.

The etiological agent of the disease is known as infectious bursal disease virus (IBDV) and belongs to the species *Avibirnavirus gumboroense*, part of the family *Birnaviridae*. It is a non-enveloped, icosahedral virus featuring a double-stranded RNA genome divided into two segments, named A and B. The 3.2 kb-long segment A contains two overlapping open reading frames (ORFs): the smaller ORF encodes a non-structural protein (VP5) involved in viral release and dissemination, whereas the larger one encodes a polyprotein that is then cleaved into the capsid protein (VP2), which self-assembles into 260 trimeric subunits to form the outer surface of the capsid; the scaffold protein (VP3), which forms its inner surface and binds the IBDV genome; and the viral protease (VP4), which constitutes the middle part of the polyprotein and is responsible for the cleavage of its own N- and C-termini and further VP2 maturation. The 2.9 kb-long segment B encodes only the viral polymerase (VP1), responsible for replication and transcription [4].

IBDV exhibits substantial genetic variability, driven by the accumulation of nucleotide substitutions, inter-segment reassortment and, to a lesser extent, homologous recombination [5]. Although two serotypes, designated 1 and 2, are recognized, and only the former is pathogenic, multiple classifications have been proposed over the years to explain the extensive differences across strains. The first isolates described in the 1960s, associated with typical disease manifestations and limited mortality, are known as classical IBDVs; a second type, which emerged in the 1980s, is antigenically related to classical viruses but responsible for more severe outbreaks with higher mortality, which led to the definition of very virulent IBDV (vvIBDV); the third historically established type, also known since the 1980s, groups the so-called variant IBDVs, which are antigenically divergent from both classical and vvIBDVs and cause subclinical infections characterized by rapid bursal atrophy and strong immunosuppression [6,7]. Such distinctions are based on the determination of both antigenicity, assessed using panels of monoclonal antibodies (mAbs) or virus neutralization (VN) assays, and pathogenicity, which requires standardized in vivo challenge trials.

While still essential, these approaches present several limitations, including their difficult implementation in routine diagnostics, challenging standardization, and limited comprehensiveness, which have become increasingly evident with the description of numerous atypical IBDV types over the years [8,9,10,11,12]. Conversely, the widespread adoption of molecular techniques has provided a convenient and easily standardized platform for the high-resolution characterization of genetic variants which, when coupled with the proposal of phylogeny-based classification criteria [13,14,15], enables a deeper understanding of IBDV heterogeneity.

The considerable variability among IBDV strains is not only relevant for strain discrimination, but it also has important practical repercussions: as previously mentioned, different IBDV types may elicit disease forms ranging from acute outbreaks with high mortality to subclinical infections associated with insidious immunosuppression; antigenically divergent strains may escape the immunity provided by vaccines based on established isolates; the occurrence of specific genetic changes may facilitate IBDV attenuation and adaptation to different hosts and cell cultures, which are essential for vaccine development and viral isolation; moreover, establishing reliable markers associated with pathogenicity or specific antigenic features may represent a valuable diagnostic resource.

Research efforts aimed at understanding the molecular basis of IBDV functional features began more than 30 years ago [7], and a wealth of data is available on this subject. Specific regions of IBDV genome have long been recognized as important determinants of its phenotype and are routinely targeted for strain characterization, obviating the need for functional assays. However, the reliance on studies focusing on the effect of specific amino acid residues is currently hindered by a lack of systematic organization: different studies may have focused on different sites, investigated under heterogeneous experimental conditions and in diverse epidemiological contexts, preventing direct comparisons and potentially contributing to the propagation of biases due to the continuous use of outdated or poorly supported markers.

For this reason, the aim of the present study was to provide a systematic overview of which amino acid sites have been identified as relevant determinants of IBDV antigenicity and pathogenicity based on the available scientific literature.

## 2. Materials and Methods

### 2.1. Eligibility Criteria

This scoping review aimed to retrieve all original research articles providing experimental evidence (in vivo, in vitro or in silico) on the role of specific amino acid positions or amino acid changes for the determination of IBDV antigenicity or pathogenicity.

Functionally relevant epitopes were also included when their length was considered sufficiently informative to reflect localized antigenic or functional determinants. A cutoff of 25 amino acids was applied to exclude extended regions likely encompassing multiple determinants and therefore limiting interpretability, while still allowing the inclusion of shorter epitopes commonly identified through peptide-based approaches.

Other publication types (i.e., reviews, book chapters, preprints, gray literature, etc.) and articles written in languages other than English were not considered. Studies based solely on observational or epidemiological associations were excluded to avoid weak causality, limit redundancy from repeatedly reported positions and to ensure consistent evaluation of functionally supported evidence.

### 2.2. Search Strategy

Dedicated queries (Supplementary Table S1) were built to search three databases, namely PubMed [16], Scopus [17], and Web of Science Core Collection [18], which were interrogated on 15 July 2025. The retrieved references were imported into the Rayyan web platform [19] for automated duplicate removal. To identify newly published studies, the search was updated on 25 March 2026 using the same strategy and databases. Neither backward nor forward citation tracing was conducted.

### 2.3. Screening

After manually retrieving the respective full texts, entries were independently screened by two out of four potential reviewers (F.R.T., F.P., C.A., M.L.) following a two-step process: first, studies were preliminary checked for any mention of specific amino acid positions; then, eligibility was further assessed by investigating whether said positions were correlated to effects related to antigenicity or pathogenicity, and sources that only reported observational evidence were discarded. In case of conflicts in any of the two steps, a third reviewer was consulted for the final decision.

### 2.4. Data Extraction

Data extraction was conducted using a structured two-step process. In the first step, one of three reviewers (F.R.T., F.P., or C.A.) independently extracted relevant data from each eligible study. A standardized form was used to retrieve the following outputs of interest:

- POSITION: amino acid position(s), amino acid change(s) or residue(s), and involved viral protein(s).
- EFFECT: effect type (antigenicity or pathogenicity) and effect description.
- EVIDENCE: evidence type (in vivo, in vitro, in silico) and implemented method(s).
- EPIDEMIOLOGY: involved viral type and corresponding genotype. Genotypes were assigned according to the classification proposed by Islam et al. [14], which is based on the combined characterization of genome segments A and B. In this framework, the term ‘genogroup’ refers to the classification of a single segment (i.e., A1 or B1), whereas ‘genotype’ refers to the classification of both segments (i.e., A1B1). In accordance with the original classification [14], when sequence information for one segment was unavailable, the corresponding genogroup was denoted as ‘x’ (e.g., A2Bx).

In the second step, an expert reviewer with domain expertise (M.L.) performed a comprehensive re-evaluation of all eligible studies and extracted data, ensuring consistency and accuracy across the dataset. This validation step included the verification of amino acid positions, confirmation of functional interpretations, and harmonization of genotype assignments.

As VP sequences from different IBDV strains may exhibit minor length variability, a standardized numbering scheme was adopted based on the predominant annotation available in the UniProt database [20], using isolate Chicken/Cuba/Soroa/1998 (taxon ID: 645118) as a curated representative sequence. Within the considered polyprotein reference (1012 aa; GenBank accession no. AAD30136), VP2 spans residues 1–441, VP4 spans residues 513–755, and VP3 spans residues 756–1012. All analyses and residue numbering reported herein refer to mature protein coordinates, specifically VP2 (441 aa), VP4 (243 aa), and VP3 (257 aa). VP1 (879 aa; GenBank accession no. ABS18957) and VP5 (145 aa; GenBank accession no. P0C751) were treated analogously.

### 2.5. Evidence Synthesis and Reporting

The retrieved data were exported from Rayyan and subjected to qualitative evidence synthesis, which was performed in Microsoft Excel (ver. 2602). To facilitate interpretation, the collected evidence was stratified based on the degree to which individual studies allowed unambiguous attribution of antigenic or pathogenic effects to specific amino acid positions. Considering the evidence type, implemented methodology, and extent to which alternative explanations (i.e., concurrent changes in other positions, unaccounted regions or viral proteins) were excluded, studies were divided into three levels of evidence:

- DIRECT ATTRIBUTION was assigned when all the following criteria were met: (i) the phenotype was directly assessed using in vivo or in vitro assays; (ii) the effect of individual amino acid substitutions was evaluated; and (iii) the genetic background was controlled (i.e., reverse genetics or equivalent systems), such that no additional concurrent amino acid changes affecting the phenotype could be present. For antigenicity studies, mAb-based assays targeting defined antigenic regions were considered eligible, as altered mAb recognition constrains the effect to the mapped epitope; studies were classified as direct attribution when the observed effect could be attributed to a single substitution within the epitope.
- MULTI-SITE ATTRIBUTION was assigned when: (i) the phenotype was directly assessed using in vivo or in vitro assays; (ii) multiple amino acid substitutions or defined epitopes were evaluated, preventing isolation of the effect of individual residues; and (iii) the genetic background was controlled (i.e., reverse genetics, engineered constructs, or epitope mapping), such that no additional concurrent amino acid changes affecting the phenotype were present or unaccounted for. For antigenicity studies, mAb-based assays targeting defined antigenic regions were considered eligible when altered recognition could be attributed to a defined epitope but involved multiple concurrent substitutions.
- ASSOCIATIVE EVIDENCE was assigned when at least one of the following conditions applied: (i) the genetic background was not controlled, such that additional substitutions or genomic regions potentially contributing to the phenotype could not be excluded (i.e., partial-genome analyses or passaging studies without full-genome characterization); or (ii) the evidence was derived solely from in silico analyses.

Visual representations of relevant positions within the different viral proteins were generated using R 4.4.1 [21]. The Preferred Reporting Items for Systematic reviews and Meta-Analyses extension for Scoping Reviews (PRISMA-ScR) Checklist [22] was adopted to ensure reporting data collection completeness, analysis, or interpretation.

## 3. Results

### 3.1. Scoping Review Process

**Figure 1:**
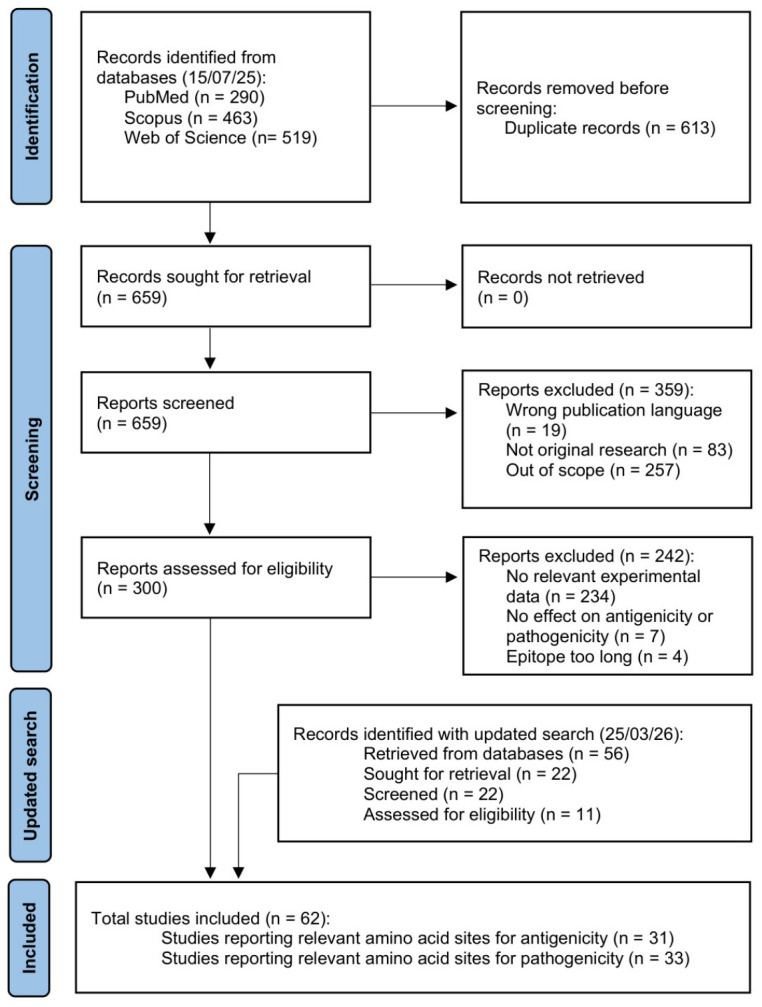
Flow diagram of the study selection process, adapted from the PRISMA 2020 template [23] in accordance with PRISMA-ScR guidelines [22].

A total of 1272 studies were retrieved from the three databases, of which 613 were duplicates. Out of the 659 screened entries, 359 were excluded in the preliminary screening phase whilst 300 were subjected to final eligibility assessment. After removing 242 publications that did not contain relevant information, 58 articles underwent data extraction. The updated search identified 56 additional records, of which 34 were duplicates. Following the removal of 11 entries during the preliminary screening and 7 during final eligibility assessment, 4 new records were subjected to data extraction, bringing the total number of included studies to 62. At the end of the review process (summarized in Figure 1), data on relevant sites for antigenicity and pathogenicity determination were obtained from 31 and 33 articles, respectively.

### 3.2. Molecular Determinants of Antigenicity

A total of 42 amino acid positions and 7 epitopes across the different VPs encoded by segment A have been reported to contribute to antigenicity determination, either individually or in combination, as listed in Table 1.

**Figure 2:**
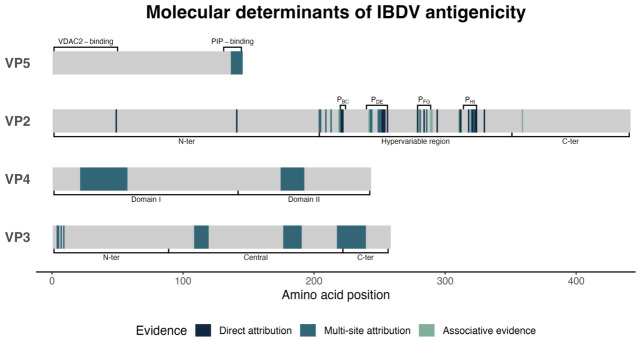
Molecular determinants of antigenicity across relevant viral proteins and respective domains. Amino acid positions were color-coded based on the level of associated evidence. Direct attribution indicates effects unambiguously linked to individual residues; multi-site attribution indicates effects attributed to defined epitopes or sets of residues without resolution of individual contributions; associative evidence indicates correlations without confirmed causal relationships. P_BC_, P_DE_, P_FG_, P_HI_: hydrophilic loops of the projection domain; PIP: phosphoinositide; VDAC2: voltage-dependent anion channel 2.

Most evidence on IBDV antigenicity was produced in vitro, primarily looking at alterations in mAbs recognition by antigen capture (AC)-ELISA or VN assays. In silico results, obtained with structural modeling and antigenic indexing approaches, were less frequent and mostly ancillary, and only little in vivo research demonstrated direct effects on immunogenicity or breakthrough power in immunized chickens. Although many studies focused on specific regions of the VP2, the frequent reliance on reverse genetics platforms allowed unambiguous associations between the introduced amino acid substitutions and the resulting changes. Similarly, the antigenically relevant positions within VP3, VP4 and VP5 were identified by peptide scanning which, despite unable to pinpoint single positions, ensured that the observed effects were independent from any change involving the VP2. Figure 2 shows the position of antigenic determinants across the different VPs, along with the level of associated evidence, which is also detailed in Supplementary Table S2.

#### 3.2.1. Antigenic Determinants Within VP5

The antigenic relevance of VP5 is supported by a single study, in which an epitope was identified towards the C-terminus of the protein (aa 137–145). Such epitope, well conserved across serotype 1 strains, was shown to elicit highly specific mAbs that could discriminate between wild-type and VP5-deleted IBDVs [25].

#### 3.2.2. Antigenic Determinants Within VP2

The contribution of different VP2 residues to antigenicity determination has been extensively researched, with most positions being located within its hypervariable region (HVR). More precisely, they are concentrated in the hydrophilic loops corresponding to the most exposed parts of the projection domain of the VP2 [55], which would explain their functional relevance.

Although residues are mostly found in specific combinations and work together to define the antigenic profile of IBDV strains, the individual effect of some sites is well-established. One of the best studied amino acid sites is position 222: in general, classical strains (A1B1) feature a proline, whereas alanine is mostly reported in vvIBDVs (A3B2), threonine and glutamine in antigenic variants (A2B1), and serine in distinct IBDVs (A4B1) [27,32,33,35,36,38]. Another highly variable residue is located at position 254, where any changes from glycine (including asparagine, serine, and aspartic acid) contribute to antigenic divergence from vvIBDVs (A3B2) [30,32,38,43,46,48]. Similarly, the alanine at position 321, that is commonly seen in A3B2 strains, is often substituted with different amino acids (threonine, valine, glutamic acid, or aspartate) in other genotypes or antigenically atypical strain [26,27,29,35,36,39,43,46]. Additional substitutions have been specifically associated with the divergence of antigenic variants and to the possible circumvention of the protection induced by classical-based vaccines, including I256V [42,48], D279N [42,47], G318D/N and D323E/Q [25,26,29,35,37,42,45,50].

Among antigenically relevant sites, only three are outside the HVR: changes from threonine to alanine at position 49 and from tyrosine to histidine at position 141, located in the N-terminal domain and part of the shell subunit of the VP2, were shown to individually determine the loss of reactivity with specific mAbs [37]; as for site 359, located in the C-terminal domain and also included in the shell subunit, the presence of a lysine was predicted to contribute to the divergent antigenic profile of distinct IBDVs (A4B1) isolates compared to other genotypes based on in silico analyses [41].

#### 3.2.3. Antigenic Determinants Within VP4

Wang et al. [51] identified three VP4 portions as T cell epitopes. More specifically, two epitopes, spanning aa 22–39 and 40–57, are recognized only by CD8+ T cells, whereas a third one (aa 175–192) is recognized by both CD4+ and CD8+ T lymphocytes. Following experimental infection with a recombinant fowl adenovirus strain expressing the purified VP4, the elicited cellular immunity proved protective against IBDV challenge [51].

#### 3.2.4. Antigenic Determinants Within VP3

Several VP3 portions were established as antigenic determinants: Saravanan et al. [52] located an epitope towards the C-terminus of the protein (aa 218–239) that elicited mAbs capable of detecting IBDV in clinical samples when used in an ELISA test; similarly, Deng et al. [53] reported two fairly conserved and strongly immunogenic peptides at positions 109–119 and 177–190; lastly, Pan et al. [54] determined that residues at positions 4, 5, 7 and 9 are critical for recognition by a mAb with neutralizing effects.

### 3.3. Molecular Determinants of Pathogenicity

A total of 57 amino acid positions and a single epitope across all viral proteins have been associated with changes in pathogenicity, either individually or in combination, as listed in Table 2.

**Figure 3:**
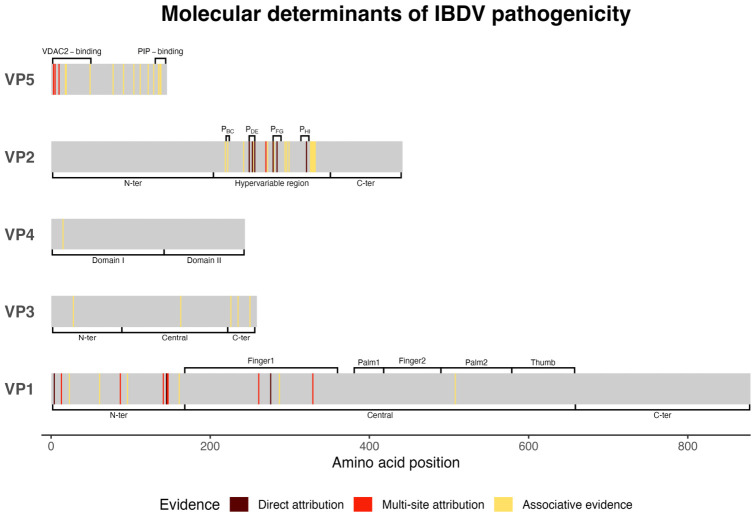
Molecular determinants of pathogenicity across relevant viral proteins and respective domains. Amino acid positions were color-coded based on the level of associated evidence. Direct attribution indicates effects unambiguously linked to individual residues; multi-site attribution indicates effects attributed to defined epitopes or sets of residues without resolution of individual contributions; associative evidence indicates correlations without confirmed causal relationships. P_BC_, P_DE_, P_FG_, P_HI_: hydrophilic loops of the projection domain; PIP: phosphoinositide; VDAC2: voltage-dependent anion channel 2.

Pathogenicity determinants have been mostly studied in vivo, usually by evaluating their influence on mortality, clinical signs, bursal atrophy or lesions following inoculation. The changes of interest could have been introduced by reverse genetics, allowing an unambiguous association with the observed effects, or observed after serial passaging in embryonated eggs or cell cultures, in which case the level of evidence was often limited by the fact that only a part of IBDV genome was considered, not allowing to exclude undocumented substitutions. Studies establishing markers of pathogenicity by sequence comparison or other indirect assessments, which often did not consider the entire IBDV genome, were considered as a lower level of evidence (Figure 3, Supplementary Table S3).

#### 3.3.1. Pathogenicity Determinants Within VP5

Multiple amino acid positions within VP5 were associated with changes in pathogenicity: Wang et al. [56] reported the occurrence of a set of simultaneous mutations (F18L, R49G, F78I, E91G, G104C, Y122H, P129S, W137R) during serial cell culture passaging of vvIBDV Gx strain (A3B2) that led to a drop in mortality and bursal lesions; Ren et al. [57] noted three nearby substitutions (T135I, W137R, and H138N) in a naturally attenuated vvIBDV (A3B2); Hernández et al. [58] found two highly conserved positions (49R and 137W) in vvIBDVs (A3B2); Mató et al. [59] observed two changes (N19D A112V) in a very virulent/classical attenuated reassortant (A3B1) causing no mortality and reduced bursal lesions compared to traditional vvIBDVs (A3B2). Whilst some of these findings overlap, strengthening their significance, none of these studies allowed to isolate the effect of VP5 changes from those of concurrent substitutions in other viral proteins.

By relying on a reverse genetics approach, Gao et al. [60] produced more robust evidence on the involvement of specific VP5 sites in pathogenicity determination, linking S3A, S5G and R10A to the attenuation of a classical IBDV strain (A1B1) and thus to decreased apoptosis and viral replication in DF-1 cells as well as reduced bursal atrophy and lesions in SPF chickens.

#### 3.3.2. Pathogenicity Determinants Within VP2

Extensive evidence has been produced on the pivotal role of the capsid protein for pathogenicity determination. Although such effect has been attributed to many different residues, all located within the HVR, some of them were studied more and are currently interpreted as reliable markers of pathogenicity.

The earliest example in this regard is the SWSASGS motif at positions 326–332, observed by Heine et al. [25] in sequences of highly pathogenic strains. Conversely, low or non-pathogenic strains feature one or two serine changes that possibly alter the interactions involving this region. Nonetheless, it should be noted that this marker, which is still frequently referenced for pathogenicity inference, was established in silico, and the actual effect of the included positions was never corroborated by subsequent studies.

Another relevant amino acid is located at position 253: an H → G change, acquired by classical attenuated vaccines (A1B1) when serially passaged in chickens, was shown to increase their virulence [61]; a similar effect was linked to the H → Q substitution when occurring, either alone or in combination, in classical (A1B1) [71,79], very virulent (A3B2) [68,76] and antigenic variant strains (A2B1) [67]; conversely the Q → H leads to attenuation in A3B2 [65,69,73,74] and A2B1 IBDVs [42]. Li et al. [76] attributed this functional relevance to conformational changes, with glutamine enabling a stronger binding to IBDV-specific receptors and enhancing the pathogenic potential.

Concurrent changes, albeit with an independent effect, are frequently observed at position 284, with alanine and threonine being associated, respectively, to increased and decreased virulence [42,64,65,67,68,69,73,76]. Notably, residues 253 and 284 are located, respectively, at the peak of the P_DE_ (aa 249–256) and P_FG_ (aa 279–289) hydrophilic loops, constituting exposed parts of the projection domain [55]. Together with nearby position 279, where a D → N substitution was shown to contribute to the attenuation of different pathotypes [47,64,66,69], these sites are all suspected to interact to target cell receptors [55], which would explain their major influence on pathogenicity.

Several amino acid mutations in nearby positions could also play an important role: among the best characterized are Q249R, I256V, A270T/E and I272T, which may separately lead to attenuation of vvIBDVs (A3B2) [47,63,66,72,75,80]. A222P and A321V changes, located at the peak of the P_BC_ (aa 220–224) and P_HI_ (aa 314–324) loops, were also demonstrated to decrease virulence [39]. In particular, the effect of the latter modification was hypothesized to stem from the alteration of local conformation, which in turn may modify the interactions involving the different VP2 loops [39,69].

#### 3.3.3. Pathogenicity Determinants Within VP4

The involvement of VP4 in pathogenicity determination has been suggested by a single article [72], which reported a P → T substitution at position 75 (corresponding to position 527 within the polyprotein) following the attenuation of vvIBDV ks strain (A3B2) through serial passaging on SPF eggs, which resulted in the complete absence of mortality and clinical signs. However, it should be noted that concurrent substitutions were observed in the VP2 and VP1.

#### 3.3.4. Pathogenicity Determinants Within VP3

VP3 variation was never demonstrated to directly affect pathogenicity, but Wang et al. [56] observed five amino acid substitutions (H28Q, E163A, P226L, A235V, A250T) during serial cell culture passaging of vvIBDV Gx strain (A3B2) that corresponded to a significant decrease in mortality and bursal lesions.

#### 3.3.5. Pathogenicity Determinants Within VP1

Several VP1 residues were reliably identified as pathogenicity determinants: for instance, multiple studies proved the correlation between a valine at position 4 and the pathogenic potential of vvIBDVs (genogroup B2), and that a change to isoleucine, either alone or in combination, leads to a significant decrease in mortality, bursal atrophy and lesions [57,83,85]. Another frequently referenced marker of pathogenicity is found at positions 145–147, with vvIBDVs (A3B2) displaying either a TEG or TDN triplet and non-vvIBDVs having a NEG triplet [84]. The attenuating effect was attributed to either T145N and D146E + N147G, which decreased pathogenicity either alone or synergistically [84]. However, it should also be noted that Yu et al. [83] failed to confirm the relevance of T145N as well as another tentative marker at position 61 [82].

Additional single (A276T, T329A) or combined substitutions (K13T + L141V, D146E + N147G) were associated with variable levels of attenuation when introduced through reverse genetics [39,80,84,85]. Other changes possibly associated with similar effects were also noted in naturally atypical (A3B2) [57], serially passaged (A3B2) [72], or reassorted (A3B1) [59] strains.

Aside from vvIBDVs, the introduction of two substitutions (R87Q and L261P) in an antigenic variant strain backbone (A2B1) enhanced the severity of bursal lesions [67], suggesting that different amino acids may be functionally important for different viral types.

## 4. Discussion

The results of the evidence synthesis highlight the complexity of the determination of IBDV phenotype, which is influenced by multiple amino acid positions located across different VPs.

A significant portion of functionally relevant residues has been identified within the VP2, which is pivotal for both antigenicity and pathogenicity. Its exposure on the viral surface makes it the primary target for neutralizing antibodies, which are considered the main correlates of protection [35,86]. Functionally relevant residues are predominantly located within the HVR, particularly in exposed regions of the projection domain. While major antigenic sites cluster within the hydrophilic loops, accumulating evidence indicates that adjacent regions also contribute to antigenic variation.

VP2 contributes to IBDV pathogenesis by inducing the apoptotic process that leads to lymphocyte B depletion [87]. Such pro-apoptotic effect likely depends on different pathways: in particular, the interaction between VP2 and Oral Cancer Overexpressed protein 1 (ORAOV1) leads to an increase in reactive oxygen species (ROS) production, which in turn promotes the release of cytochrome c from the mitochondria to the cytosol [88]. Moreover, VP2 directly interacts with Programmed Cell Death protein 1 (PD-1), a host immunoinhibitory factor primarily expressed on the surface of activated lymphocytes, thus suppressing the anti-apoptotic phosphoinositide 3-kinases/protein kinase B (PI3K-AKT) signaling cascade [89].

Additionally, VP2 is involved in viral tropism determination, as its projection domain is critically involved in the attachment to specific cell surface receptors: in particular, the conserved ^234^IDA^236^ triplet was shown to bind to α4β1 integrin [90], which is abundantly expressed on the surface of immature B lymphocytes [91], triggering an actin rearrangement cascade that results in viral entry into the cell via endocytosis [92]. Although the overall attachment and entry processes have not been fully elucidated, several other components of the receptor complex were also proposed to promote viral infection through VP2 interaction, including the λ light chain of surface immunoglobulins M (IgM), CD74 and CD44 proteins expressed by B-cells [93,94,95], as well as the chicken heat shock protein 90 (Hsp90) and heat shock cognate protein 70 (HSC70) found on DF-1 chicken fibroblasts [96].

Viral tropism and pathogenicity are evidently linked, as tissue culture adaptation typically leads to strain attenuation [64,65,69,77], whereas in vivo passaging of cell-adapted strains often results in pathogenicity enhancement and reversion to virulence [61,64,68]. Notably, the VP2 residues involved in these processes seem even more clustered than antigenically relevant ones, with no positions being identified outside the HVR and most being concentrated within the hydrophilic peaks.

Although VP2 historically received the most attention, other viral proteins have long been recognized as crucial determinants of pathogenicity and antigenicity. For instance, the involvement of VP5 in IBDV pathogenesis was already demonstrated by Yao et al. [97], who observed that a modified strain lacking this non-structural protein showed decreased cytotoxic and apoptotic effects in cell culture and failed to produce any sign or lesion in vivo. Further studies confirmed that VP5, although not required for replication [98], is involved in apoptosis induction [99,100]. However, the exact function played by VP5 depends on the stage of infection: in the early stages, VP5 inhibits apoptosis by activating PI3K/AKT signaling pathway, allowing for viral replication [101,102]; at later stages, it promotes apoptosis to favor viral release and dissemination by interacting with the voltage-dependent anion channel 2 (VDAC2) in the mitochondria of the host cells, enhancing caspase activation and cytochrome c release [103]. The apoptotic mechanism appears both self-sufficient and more potent than the analogous action exerted by the VP2 [103].

VP5 tends to be conserved within the same serotype, while a more significant divergence is observed between serotype 1 and 2. As a matter of fact, replacing the VP5 gene of a vvIBDV with that of a serotype 2 strain decreased its cytotoxicity, supporting the crucial contribution of this viral protein to the different pathogenicity of the two serotypes [104].

Residues linked to pathogenicity determination appear distributed across VP5, but some of the best-established ones are located towards the N-terminus and within the VDAC2-binding domain (aa 1–50), supporting the hypothesis that their substitution may lead to differences in cell damage potential [60]. Similarly, the domain that binds membrane-bound phospholipids known as phosphoinositides (PIPs) and accounts for VP5 tropism for plasma membrane, located at the C-terminus (aa 131–145), also contains some sites whose variability was linked to decreased virulence, albeit together with concurrent substitutions [56,57,58].

Another interesting VP5 feature is the existence of a possible upstream alternative AUG start codon, which adds a 4 amino acid-long extension to the N-terminal region and brings VP5 length to 149 rather than 145 amino acids [105]. Whilst originally observed in vvIBDVs and not in other genotypes [105,106,107], such extension was never associated with pathogenicity determination, and its absence in some vvIBDVs showing typical pathogenicity supports this hypothesis [58].

The antigenic role of VP5 was noted by Mundt et al. [98], who reported that the absence of VP5 expression resulted in a lack of reactivity with either anti-VP5 mAbs and polyclonal sera. Han et al. [25] later identified an epitope in the PIP-binding domain, although not directly involved in plasma membrane targeting. Nonetheless, such changes appear more relevant for diagnostic purposes (i.e., by potentially coupling them with VP5-deleted vaccines) than for actual immunity.

While VP1 has no implications for antigenicity, its pivotal role in pathogenicity determination is clearly demonstrated by many reports of naturally occurring or reverse-engineered reassortants displaying differences in pathogenicity compared to their progenitors sharing the same segment A [39,108,109]. Being VP1 an RNA-dependent RNA polymerase (RdRp), the biological reasons behind its influence on pathogenicity are not as immediate as for VP2 and VP5, especially considering that this trait was demonstrated to be independent from replication efficiency [110]. However, it is worth noting that IBDV VP1 shows a greater mutation rate than the RdRps of analogous viruses, suggesting that its variability may contribute to viral fitness [5,111].

A trend can be identified by looking at the position of relevant amino acids within the VP1: the majority of the substitutions which were experimentally linked to pathogenic changes are concentrated within the N-terminal region (aa 1–168) of the VP1 [112], that is responsible for the distinctive birnavirus feature of RNA synthesis initiation through self-guanylation [113], and within the first finger subdomain (aa 168–360), involved in template and nucleoside triphosphates binding [114]. Since these regions are linked to replication fidelity, said modifications were hypothesized to support the generation of a more variable progeny [5], potentially affecting the pathogenic potential. In comparison, the C-terminal region of the VP1 appears far more conserved, likely due to functional constraints [111].

The available evidence reporting phenotypically relevant residues across VP3 and VP4 was comparatively sparser, likely due to their higher degree of conservation but also to the lesser focus on these VPs. IBDV VP3 performs many essential functions: it coordinates capsid morphogenesis by interacting with the VP2 [115] and ensuring the proper encapsidation of the VP1 [99]; it binds to dsRNA molecules, forming thread-like ribonucleoprotein complexes [116], and inhibits antiviral innate immunity by blocking viral RNA recognition pathways [117,118]; since the dsRNA is a potent activator of protein kinase R (PKR), its binding to VP3 also has anti-apoptotic effects, counteracting the action of the VP2 until the virus is accumulated and ready for release [119]; lastly, VP3 actively participates in replication, recruiting the VP1, stimulating its activity and acting together to compensate the lack of a 5′ cap typical of IBDV genome, thus enabling translation initiation [120,121]. Most of the required interactions involve the C-terminal region, where the occurrence of a specific change was shown to impact replicative functions [122].

Since VP3 forms the inner, unexposed layer of the capsid, its antigenic relevance is not on a par with the VP2. Nonetheless, the existence of cross-reactive or serotype-specific VP3 epitopes has been long established [123,124] and traced to different domains [125,126,127]. Subsequent studies were able to locate two epitopes in the central region [53] and one towards the C-terminus [52]. Moreover, Pan et al. [54] identified four highly conserved residues in the N-terminal domain that could be specifically recognized by a neutralizing mAb.

VP4 is a serine protease involved in viral maturation, cleaving the polypeptide and processing the VP2 from its precursor to final form [128]. Together with the VP3, it also contributes to the inhibition of innate antiviral responses, acting as an interferon suppressor by interaction with the glucocorticoid-induced leucine zipper (GILZ) [129]. Wang et al. [51] identified two contiguous immunogenic epitopes within Domain I, which houses the substrate groove and specificity pockets, and another in Domain II, which contains the serine-lysine catalytic dyad [130]. All these conserved epitopes are recognized by T cells and were shown to induce specific and protective cellular immunity.

Even if amino acid positions potentially associated with pathogenicity have been reported in both VP3 and VP4, their actual relevance remains disputable, as the only two available studies also identified concurrent and more established changes in VP1, VP2 and VP5 [56,72]. Therefore, such evidence should be interpreted with caution and would benefit from further validation.

Aside from identifying functionally relevant positions, this scoping review highlights the substantial heterogeneity in the research approaches used to assess their role. Although almost all studies focusing on antigenicity were conducted in vitro, their comparability was limited by clear differences in experimental design (i.e., different assays, mAbs, reference sequences, etc.). The same applies to pathogenicity studies, which were not always easy to generalize to field conditions despite often including in vivo experiments.

The most common limitation was the frequent focus on restricted genomic regions, particularly the VP2 HVR, which does not only affect the robustness of the findings but also contributes to the underrepresentation of other VPs despite their long-recognized relevance. Similarly, experiments have been predominantly conducted on well-characterized IBDV types, often using a limited number of isolates. Most antigenicity studies focus on strains belonging to segment A genogroups A1, A2, and A3, with only a few addressing more divergent but established genogroups such as distinct (A4) [32,36] and Early Australian (A7) [31] IBDVs. Likewise, pathogenicity studies, which should ideally account for both genome segments to capture the contribution of VP1, have historically focused on classical (A1B1), antigenic variant (A2B1) and vvIBDVs (A3B2), with only a limited number of recent investigations exploring emerging reassortants such as A3B3 [81] and A3B1 [59] as well as novel variant IBDVs (A2B1) [78]. This uneven distribution of evidence reflects, at least in part, the more recent identification of several genotypes, their perceived lower relevance, or their circulation in under-sampled geographical regions. However, the limited functional characterization of these viruses hinders the accurate assessment of their pathogenic and antigenic properties, thus increasing the risk of misclassification, hampering their early identification, and possibly affecting surveillance and control efforts.

Notably, a 25-amino-acid cutoff length was adopted for epitope inclusion as a pragmatic compromise between resolution and inclusiveness: on one hand, it allowed the incorporation of relevant evidence for proteins primarily characterized through peptide mapping, but on the other it led to the exclusion of a small number of early studies [123,124,125,126] describing larger VP3 portions. Such regions, although potentially relevant, were deemed less informative for the identification of discrete molecular determinants, which were more precisely detailed by later studies.

Another choice that should be mentioned was to exclude purely observational studies. While this approach was intended to prioritize functionally supported markers and reduce redundancy from repeatedly reported sites, it may underrepresent amino acid positions consistently correlated with phenotypic outcomes in field settings. Consequently, some markers commonly used in epidemiological surveillance may not have been captured in the present dataset.

From an applied perspective, it is important to distinguish between amino acid markers used for molecular surveillance and those with demonstrated functional relevance. Routine epidemiological classification of IBDV strains should primarily rely on phylogenetic analysis, which provides a more robust framework for characterization than individual residue-based interpretation. In this context, the positions identified in this review may help generate hypotheses regarding pathogenicity, immune escape, or vaccine performance, but they should not be intended as standalone diagnostic markers.

Although agreement among the retrieved studies was generally good, notable inconsistencies were identified. For instance, the D279N substitution, whose functional relevance is frequently reported and supported by reverse genetics experiments [28,47,62], has been shown in other studies to have no effect on pathogenicity, either individually [131] or in combination with another well-established determinant such as A284T [132]. These discrepancies have been attributed to the influence of the broader genomic background, as the effect of a given residue may depend on the presence of additional substitutions within the same protein or in other viral components [47]. These observations suggest that molecular markers should not be interpreted in isolation, but rather within the broader genetic context in which they occur. Practically, their application should therefore consider the level of supporting evidence, the involved viral genotype and epidemiological situation, and the co-occurrence of additional amino acid changes with potential synergistic or compensatory effects.

Beyond the interpretation of existing evidence, several recommendations can be proposed to improve future studies and enhance the reliability and comparability of results: investigations should aim to be either as comprehensive as possible (i.e., incorporating full-genome sequencing to capture all relevant regions) or highly targeted (i.e., using reverse genetics approaches to exclude confounding factors), and sequence comparisons should be broad and systematic, rather than relying on a limited set of arbitrarily selected reference sequences. In practice, residues and regions supported by lower-tier evidence (i.e., multi-site or associative), particularly when identified across multiple independent studies and genomic backgrounds, would represent the obvious candidates for experimental validation in controlled genetic systems, that would help clarify their individual contribution and potential context-dependent effects. Moreover, reporting should be transparent and standardized, with detailed descriptions of experimental conditions and measured outcomes, and results should be discussed in the context of the full body of available evidence rather than selectively referencing concordant studies.

While molecular determinants are widely acknowledged in research contexts, it is also important to consider how these findings translate into diagnostic and control strategies. Currently, dual-segment phylogenetic analyses represent the standard for IBDV classification [14,15], primarily due to the need to account for the role of VP1 and reassortment events in pathogenicity. Although these approaches capture most of the established markers, they remain focused on the VP2 HVR and the VP1 B-marker region, thereby excluding potentially relevant determinants located in other genomic regions.

From a practical perspective, these criteria represent an effective compromise between informativeness and applicability in routine diagnostics. However, caution is warranted when inferring phenotypic traits based solely on partial sequences, as important determinants outside the considered VP2 and VP1 portions may be overlooked. In this context, the expansion of sequencing efforts to additional genomic regions may improve the reliability of genotype–phenotype inference while remaining compatible with routine workflows. Such an approach would also help addressing the current underrepresentation of other VPs in the literature and public databases, that likely contributes to an underestimation of their functional relevance. More specifically, the inclusion of VP5 sequencing, which can be achieved through assays that simultaneously target the VP2 [58], may provide added value when assessing pathogenicity. Similarly, the presence of immunogenic epitopes in VP3, VP4, and VP5 suggests additional opportunities for serological characterization: although antigenicity is primarily driven by VP2, these less-characterized regions may represent promising targets for the development of serodiagnostic tools aimed at discriminating between strains and between infection- and vaccine-induced immune responses [25,133,134].

Regarding control strategies, a deeper understanding of the role of specific amino acid residues provides a framework for the rational design of improved vaccines, particularly through reverse genetics approaches rather than traditional attenuation methods [135]. Several studies have linked reduced vaccine efficacy to specific HVR residues in vvIBDVs (A3B2) [47], as well as in traditional [30,38,40] and emerging antigenic variants (A2B1) [42,45], highlighting positions such as 222, 254, 279, 318, and 323 as key targets for sequence optimization. These findings support the use of molecularly informed antigen design to improve cross-protection against antigenically diverse strains.

In addition, it should be noted that currently available vector vaccines rely exclusively on VP2 expression. While this strategy is both justified and effective, increasing evidence that protective epitopes may also be present in other VPs, particularly VP3 [54] and VP4 [51], suggests that incorporation of additional antigens in engineered vaccines could enhance the breadth and robustness of the immune response.

## 5. Conclusions

This scoping review synthesizes over 35 years of evidence on the molecular determinants of IBDV antigenicity and pathogenicity, highlighting the functional contribution of multiple VPs and the variable strength of support across amino acid positions. The obtained findings underscore the need for critical interpretation of molecular markers, particularly when based on partial sequences, and support the use of evidence-based frameworks for their application. Future research should prioritize the validation of lower-tier determinants in controlled genetic systems, as well as the characterization of underrepresented genotypes and genomic regions. Expanding these efforts will be essential to improve surveillance, refine diagnostic approaches, and support the rational design of next-generation vaccines.

## Figures and Tables

**Table 1:** List of amino acid positions and substitutions involved in antigenicity determination.

Residue/Substitution ^1^	Protein	Effect Description	Genotype ^2^	Evidence ^3^	Reference
^137^RRDLPKPE^145^	VP5	Specific mAb recognition	-	IVT	[24]
**G318D D323E**	VP2	Altered mAb recognition	A2B1	IVT	[25]
Q249K I286T E311K D318G Q320L A321E E323	VP2	Altered mAb recognition	A2B1	IVT	[26]
**P222S/Q/T/A A321V Q324L**	VP2	Altered mAb recognition	A3B2	IVT	[27]
**D279N A284T**	VP2	Altered mAb recognition	A3B2	IVT	[28]
D213N + Q249K + T286I + G318D + E321A	VP2	Altered mAb recognition	A2Bx A1Bx	IVT	[29]
222T + 254N + 318N + 322E	VP2	Enhanced breakthrough against mAbs elicited by classical and variant-based vaccines	A2B1	IVT	[30]
R204K + V205S + T209I P222S + T250S G243V + G244V	VP2	Altered mAb recognition and antigenic profile	A7B3	IVT IS	[31]
**P222A/S G254D/N Q324L**	VP2	Altered mAb recognition	222 254: A4Bx 222 324: A3Bx	IVT	[32]
**P222A/T Q324L**	VP2	Altered mAb recognition	-	IVT	[33]
**256I 294I**	VP2	Enable recognition by vvIBDV-specific recombinant Ab	A3Bx	IVT	[34]
**P222S/T G318D/N A321E D323E R330S**	VP2	Altered mAb recognition	-	IVT	[35]
*P222S/T A321D*	VP2	Altered antigenic profile	A4Bx	IS	[36]
**T49A Y141H I312K/T** D318N + A321E + E323D	VP2	Altered mAb recognition and antigenic profile	-	IVT IS	[37]
**T222A S254N**	VP2	Breakthrough of immunity produced by parental vaccine strain	A2B1	IVV	[38]
**A321V**	VP2	Reduced reactivity towards mAbs reacting with vvIBDVs	A3B2	IVT IS	[39]
**253E**	VP2	Earlier breakthrough in vaccinated chickens	A2B1	IV	[40]
*359K*	VP2	Altered antigenic profile	A4Bx	IS	[41]
*213N + 221K + 222T + 242V + 249K + 252I + 254N + 256V + 279N + 286I + 294L + 318D + 323E*	VP2	Altered mAb recognition	A2B1	IVT	[42]
**S222L** Y220F + G254S + A321T	VP2	Altered mAb recognition	220 + 254 + 321: A3B2 222: A3B1	IVT	[43]
S251I + D279N D279Y + G281R	VP2	Enhanced immune escape capacity	A1B1	IVT IS	[44]
**G318D D323Q**	VP2	Hampered neutralization by mAb and antiserum produced against vvIBDVs	A2B1	IVT	[45]
*Q219L + G254D + D279N + N280T* *G254D + L289P + M290I* *G254S + A321V A321E/T*	VP2	Altered antigenic relatedness with typical A1/A3 strains	-	IVT IS	[46]
**D279N**	VP2	Decreased binding and neutralization power of homologous antiserum	A3B2	IVV IVT IS	[47]
**V252I G254N I256V**	VP2	Reduced antigen–antibody affinity and interference with antiserum neutralization	A2B1	IVT IS	[48]
**Q221K**	VP2	Immune escape	A2B1	IVT	[49]
**D318G**	VP2	Altered mAb recognition	A2B1	IVT	[50]
^22^GILASPGVLRGAHNLDCV^39^ ^40^LREGATLFPVVITTVEDA^57^ ^175^SFRSTKLATAHRLGLKLA^192^	VP4	Elicitation of protective cell-mediated immune response	-	IVV	[51]
^218^KHRNPRRAPPKPKPKPNVPTQR^239^	VP3	Anti-peptide antibodies reacted specifically with IBDV	-	IVT	[52]
^109^TMGYFATPEW^119 177^PGQAEPPQAFIDEV^190^	VP3	Anti-epitope sera have good immunogenicity and epitopes are recognized by IBDV-positive serum	A3B2	IVV IVT	[53]
4F + 5K +7T + 9E	VP3	Specific recognition by neutralizing mAb	A3B2	IVT	[54]

^1^ Formatting indicates level of evidence: bold = Direct attribution; regular = Multi-site attribution; italics = Associative evidence. ^2^ According to the phylogenetic classification proposed by Islam et al. [14]. ^3^ IVV: in vivo; IVT: in vitro; IS: in silico.

**Table 2:** List of amino acid positions and substitutions involved in pathogenicity determination.

Residue/Substitution ^1^	Protein	Effect Description	Genotype ^2^	Evidence ^3^	Reference
*F18L + R49G + F78I + E91G + G104C + Y122H + P129S + W137R*	VP5	Decreased mortality and bursal atrophy	A3B2	IVV	[56]
*T135I + W137R + H138N*	VP5	Decreased mortality	A3B2	IVV	[57]
*49R + 137W*	VP5	Markers of pathogenicity	A3B2	IS	[58]
*N19D + A112V*	VP5	Absent mortality, milder bursal lesions compared to vvIBDV	A3B1	IVV	[59]
S3A + S5G + R10A	VP5	Decreased apoptosis in cell culture; decreased follicle atrophy, lymphocyte loss, and necrosis	A1B1	IVV IVT	[60]
*^326^* *SWSASGS^332^*	VP2	Marker of pathogenicity	-	IS	[25]
*H253G*	VP2	Increased bursal atrophy	A1B1	IVV	[61]
*253Q 279D 284A*	VP2	Markers of pathogenicity	A3B2	IS	[62]
*G254S + A270E*	VP2	Decreased mortality and lesions	A3B2	IVV	[63]
*D279N A284T*	VP2	Decreased (D279N)/increased (A284T) bursal atrophy and splenomegaly	A1Bx	IVV	[64]
Q253H + A284T	VP2	Decreased mortality and bursal lesions	A3B2	IVV	[65]
*I272T D279N*	VP2	Decreased mortality	A3Bx	IVV	[66]
H253Q + T284A	VP2	Increased severity of bursal lesions	A2B1	IVV	[67]
**H253Q T284A**	VP2	Reversion to virulence	A3B2	IVV	[68]
**A222P** *A222P + I242V + Q253H + I256V + D279N + A284T + I294L + S299N*	VP2	Partial (A222P) or total (full set of aa changes) attenuation of mortality and bursal atrophy	A3Bx	IVV	[69]
*242I + 256I + 294I*	VP2	Severe mortality, clinical signs and lesions	-	IVV	[70]
*H253Q*	VP2	Increased bursal atrophy and lesions	A1B1	IVV	[71]
*I272T*	VP2	Decreased mortality and clinical signs	A3B2	IVV	[72]
*S332G*	VP2	Decreased mortality	A3B2	IVV	[57]
Q253H + A284T	VP2	Decreased mortality and bursal lesions	A3B2	IVV	[73]
*Q253H + I256V + I296L*	VP2	Decreased embryo lethality	A3Bx	IVV	[74]
**A321V**	VP2	Decreased mortality	A3B2	IVV	[39]
**Q249R I256V**	VP2	Decreased bursal atrophy	A3B2	IVV	[75]
*H253Q T284A*	VP2	Stronger binding to IBDV-specific receptors	A3B2	IS	[76]
*E249Q + A270E + D279N*	VP2	Decreased cytopathic effect	A3Bx	IVT	[77]
Q253H + A284T	VP2	Decreased bursal atrophy and lesions	A2B1	IVV	[78]
*Q219L + G254D + D279N + N280T*	VP2	Absent mortality, milder bursal lesions on bursa compared to typical vvIBDV	A3B1	IVV	[59]
*249H + 253Q + 256A + 284A*	VP2	Increased bursal atrophy and lesions	A1B1	IVV	[79]
A270T	VP2	Decreased mortality and clinical signs	A3B2	IVV	[80]
**D279N**	VP2	Decreased mortality, symptoms, lesions and inflammatory response in immune organs	A2B1	IVV IVT IS	[47]
G254S Q219L + S251E + G254N + I256L + D279N + N280T + S326A	VP2	Reduced mortality	A3B3	IS	[81]
*P15T*	VP4	Decreased mortality and clinical signs	A3B2	IVV	[72]
*H28Q + E163A + P226L + A235V + A250T*	VP3	Decreased mortality and bursal atrophy	A3B2	IVV	[56]
R87Q + L261P	VP1	Increased severity of bursal lesions	A2B1	IVV	[67]
*T96N + D161A*	VP1	Decreased mortality and clinical signs	A3B2	IVV	[72]
*V4I + D146E + P687S*	VP1	Decreased mortality	A3B2	IVV	[57]
*4V + 61I + 145T + 287A + 508K + 511S + 646S + 687P*	VP1	Markers of pathogenicity	A3B2	IVV	[82]
**A276T**	VP1	Decreased mortality	A3B2	IVV	[39]
**V4I**	VP1	Decreased and delayed mortality	A3B2	IVV	[83]
**T145N** D146E + N147G	VP1	Decrease in mortality, bursal atrophy and bursal lesions	A3B2	IVV	[84]
*T23S + R511K*	VP1	Absent mortality, milder bursal lesions on bursa compared to typical vvIBDV	A3B1	IVV	[59]
T329A	VP1	Decreased mortality and clinical signs	A3B2	IVV	[80]
**V4I** K13T + L141V	VP1	Decreased mortality	A3B2	IVV	[85]

^1^ Formatting indicates level of evidence: bold = Direct attribution; regular = Multi-site attribution; italics = Associative evidence. ^2^ According to the phylogenetic classification proposed by Islam et al. [14]. ^3^ IVV: in vivo; IVT: in vitro; IS: in silico.

## Data Availability

The original contributions presented in this study are included in the article/Supplementary Materials. Further inquiries can be directed to the corresponding author.

## Supplementary Materials

The following supporting information can be downloaded at: https://www.mdpi.com/article/10.3390/v18050489/s1, Table S1: Search strings used to search different bibliographic databases; Table S2: Level of evidence associated with each amino acid position which was reported as relevant for antigenicity determination; Table S3: Level of evidence associated with each amino acid position which was reported as relevant for pathogenicity determination.
