# Supplementary material for: Mapping Molecular Determinants of Antigenicity and Pathogenicity of Infectious Bursal Disease Virus (IBDV): A Scoping Review
Source: Viruses. 2026 Apr 23;18(5):489. doi: 10.3390/v18050489 (PMC13211411; doi:10.3390/v18050489)
Supplement: Supplementary file 1 [file viruses-18-00489-s001.zip › viruses-4279450-supplementary.pdf]

Supplementary Table S1. Search strings used to search different bibliographic databases.

Database	Search string
PubMed	("infectious bursal disease virus"[All Fields] OR ibdv[All Fields]) AND ("substitution"[All Fields] OR "change"[All Fields] OR "mutation"[All Fields] OR "site"[All Fields] OR "position"[All Fields] OR "amino acid"[All Fields] OR "epitope"[All Fields] OR "domain"[All Fields]) AND ("antigenic"[All Fields] OR "antigenicity"[All Fields] OR "immunogenic"[All Fields] OR "immunogenicity"[All Fields] OR "pathogenic"[All Fields] OR "pathogenicity"[All Fields] OR "virulence"[All Fields])
Scopus	(TITLE-ABS-KEY("infectious bursal disease virus" OR ibdv)) AND (TITLE-ABS-KEY("substitution" OR "change" OR "mutation" OR "site" OR "position" OR "amino acid" OR "epitope" OR "domain")) AND (TITLE-ABS-KEY("antigenic" OR "antigenicity" OR "immunogenic" OR "immunogenicity" OR "pathogenic" OR "pathogenicity" OR "virulence"))
Web of Science Core Collection	TS=("infectious bursal disease virus" OR ibdv) AND TS=("substitution" OR "change" OR "mutation" OR "site" OR "position" OR "amino acid" OR "epitope" OR "domain") AND TS=("antigenic" OR "antigenicity" OR "immunogenic" OR "immunogenicity" OR "pathogenic" OR "pathogenicity" OR "virulence")

Supplementary Table S2. Level of evidence associated to each amino acid position which was reported as relevant for antigenicity determination. Positions are reported once and assigned to the highest level of evidence identified across studies; individual studies are therefore aggregated and not listed separately.

	Direct attribution	Multi-site attribution	Associative evidence
**VP5**		137-145	
**VP2**	49, 141, 221, 222, 252, 253, 254, 256, 279, 284, 294, 312, 318, 321, 323, 324, 330	204, 205, 209, 213, 220, 243, 244, 249, 250, 251, 281, 286, 311, 320, 322	219, 242, 280, 289 290
**VP4**		22-39, 40-57, 175-192	
**VP3**		4, 5, 7, 9, 109-119, 177-190, 218-239	

Supplementary Table S3. Level of evidence associated to each amino acid position which was reported as relevant for pathogenicity determination. Positions are reported once and assigned to the highest level of evidence identified across studies; individual studies are therefore aggregated and not listed separately.

	Direct attribution	Multi-site attribution	Associative evidence
**VP5**		3, 5, 10	18, 19, 49, 78, 91, 104, 112, 122, 129, 137
**VP2**	249, 253, 256, 279, 284, 321	270	219, 222, 242, 254, 272, 280, 294, 296, 299, 326, 327, 328, 329, 330, 331, 332
**VP4**			15
**VP3**			28, 163, 226, 235, 250
**VP1**	4, 145, 276	13, 87, 141, 146, 147, 261, 329	23, 61, 96, 135, 137, 138, 146, 161, 287, 508, 511, 646, 687
